# Palliative Psychiatry for Patients With Severe and Persistent Mental Illness: A Survey on the Attitudes of Psychiatrists in India Compared to Psychiatrists in Switzerland

**DOI:** 10.3389/fpsyt.2022.858699

**Published:** 2022-05-26

**Authors:** Julia Stoll, Anju Mathew, Chitra Venkateswaran, Anil Prabhakaran, Anna Lisa Westermair, Manuel Trachsel

**Affiliations:** ^1^Institute of Biomedical Ethics and History of Medicine, University of Zurich (UZH), Zurich, Switzerland; ^2^Department of Psychiatry, Government Medical College, Thiruvananthapuram, India; ^3^Department of Psychiatry, Department of Palliative Medicine, Believers Church Medical College Hospital, Tiruvalla, India; ^4^Mehac Foundation, Kochi, India; ^5^Clinical Ethics Unit, University Hospital of Basel (USB) and University Psychiatric Clinics Basel (UPK), Basel, Switzerland

**Keywords:** severe and persistent mental illness, ethics, psychiatry, palliative care, futility, cultures

## Abstract

**Objectives:**

Palliative psychiatry is a new approach for the care of patients with severe and persistent mental illness (SPMI) which systematically considers biological, psychological, social, and existential factors of care. To assess the attitudes of psychiatrists in India toward palliative psychiatry for patients with SPMI and to compare these to the attitudes of psychiatrists in Switzerland.

**Methods:**

In an online survey, data from 206 psychiatrists in India were collected and compared with data from a previous survey among 457 psychiatrists in Switzerland.

**Results:**

Psychiatrists in India generally considered it very important to prevent suicide in SPMI patients (97.6%). At the same time, they considered it very important to reduce suffering (98.1%) and to ensure functionality in everyday life (95.6%). They agreed that palliative psychiatry is important for providing optimal care to SPMI patients without life-limiting illness (79.6%) and considered palliative psychiatry as indicated for patients with SPMI (78.2%). By contrast, curing the illness was considered very important by only 39.8 % of respondents. Relative to psychiatrists in Switzerland, psychiatrists in India were significantly more concerned about preventing suicide and less willing to accept a reduction in life expectancy, even at the expense of quality of life in patients with severe and persistent schizophrenia and recurrent major depressive disorder. At the same time, they were significantly more likely to advocate palliative psychiatry.

**Conclusion:**

Most of the participating psychiatrists in India agreed that palliative psychiatry can be indicated for patients with SPMI. The comparison with psychiatrists in Switzerland highlights the need to take account of cultural differences in future studies of this kind. In summary, this study shows the potential of palliative psychiatry as a genuine biopsychosocio-existential approach which systematically integrates biological, psychological, social, and existential factors of care.

## Introduction

The emerging field of palliative psychiatry (or palliative care approaches in psychiatry) is increasingly attracting interest (1–9). Palliative psychiatry is based on accepting that some psychiatric symptoms are irremediable and offer a valuable means of improving the quality of life of patients with severe persistent mental illness (SPMI) [(8–11) for an in-depth discussion of the concept of SPMI]. While curative psychiatry focusses on the mental disorder with the aim of (partial) remission of symptoms, palliative psychiatry aims at improving quality of life by means other than symptom remission. Analogous to palliative care in somatic medicine, palliative psychiatry systematically considers biological, psychological, social, and existential factors of care (8). For example, a palliative care plan for a person suffering from treatment-refractory schizophrenia may include stopping clozapine (if the patient is distressed by frequent blood draws and major weight gain and experiences only low improvement of psychotic symptoms), prescribing diazepam for panic attacks due to therapy-refractory delusions, scheduling group physiotherapy (if the patient experiences a reduction of subjective stress levels after exercise and wishes to expand his social circle), providing housing where unusual behavior is tolerated (as long as it does not endanger others), scheduling regular home visits by a mental health nurse to alleviate feelings of loneliness, and offering advance care planning to ensure future care (including end-of-life care) is aligned with the wishes, values, and beliefs of the patient. Thereby, palliative psychiatry is a genuine biopsychosocio-existential approach [see (12, 13)] that includes but is not limited to end-of-life care for persons with SPMI. For detailed case examples of palliative psychiatry, please see (7, 14, 15).

When should psychiatrists apply palliative psychiatry? At what point should psychiatrists focus on palliative psychiatry in addition to curative psychiatry or on its own? In countries with relatively scarce health care resources, additional ethical difficulties may arise, and multiple factors must be considered when deciding whether to forego further treatment, including duration and severity of illness, response to previous treatment and whether it is appropriate to focus on palliative psychiatry before all other possible options have been exhausted (e.g., due to a lack of financial resources) (16). In low- and middle-income countries, where only a small fraction of mentally ill patients receive adequate psychiatric treatment (17–19), the appropriateness of palliative psychiatry is an open question.

Since the 1980s, India's Kerala region has shown how requisite palliative care can be provided free of charge, using local resources (20–25). In their descriptive study, Philip et al. (25) reported that in recent years in Kerala, patients with SPMI were commonly included in these programs. However, having a SPMI was also an important factor in early program drop out, and here as elsewhere, patients with mental illness receive insufficient health care.

However, it would be counterproductive and ethically questionable to misuse palliative psychiatry as a low-cost option for cost- and time-intensive psychiatric service users. As McGorry et al. (6) noted, an under-resourced mental health system may consign patients to persisting and unremitting illness, and we concur that no group of patients should be neglected in this way. Rather, palliative psychiatry is about abandoning harmful or ineffective treatment to focus on quality of life and reduction of suffering when further curative treatment is considered futile (6, 10, 25).

Attitudes of healthcare professionals toward palliative psychiatry for patients with SPMI might be influenced by cultural aspects and economic factors of the respective healthcare system. Therefore, in this study, the question is addressed how palliative psychiatry is understood and lived in psychiatric practice in different cultures. This survey of psychiatrists in India and its comparison with a previously published survey from Switzerland (9, 26, 27) sought to assess attitudes among psychiatrists on palliative psychiatry, especially for patients with SPMI. The comparison is especially interesting because, as discussed above, differing resource levels as well as cultural differences may promote different views of palliative psychiatry and treatment of psychiatric patients with SPMI.

## Methods

The online survey investigated attitudes among psychiatrists in India to palliative psychiatry, physician-assisted dying, and compulsory interventions for patients with SPMI. The data were then compared with findings from an earlier survey of Swiss psychiatrists using the same questionnaire (9, 26, 27). The methods used in India are described below.

### Sampling and Data Collection in India

Between April and June 2020, 3,056 members of the Indian Psychiatric Society were sent an email containing information in standard text and a survey link, followed at intervals by four reminders. In total, 562 of the recipients clicked on the link; 285 commenced the process, and 206 of these completed the questionnaire using the SoSci Survey tool. Recipients were informed that, by clicking on the supplied link, they were giving their informed consent. For reasons of data security, no record was kept of whether the questionnaire had already been processed (i.e., no IP address was saved). Only fully completed questionnaires were included in the data analysis.

### Survey and Case Vignettes

The survey and case vignettes from the corresponding previous studies were translated from the original German version to English and adapted for the use in India (9, 26–28). The adaptation and translation was done by MT, taking into account specifics of the Indian context such as the illegality of Medical Assistance in Dying. In particular, the questions on palliative sedation and physician assisted dying were removed as well as all questions and a case vignette concerning anorexia nervosa. Like the original questionnaire, the adapted version comprised 23 items, along with three additional questions on age, gender, and year of graduation from medical school (as proxy marker for career duration). Five items related to the general treatment of patients with SPMI, and eight related more specifically to palliative psychiatry and SPMI. Before answering the respective questions, to standardize the understanding of palliative care, participants were presented with the WHO definition of palliative care as “an approach that improves the quality of life of patients (adults and children) and their families who are facing problems associated with life-threatening illness. It prevents and relieves suffering through the early identification, correct assessment and treatment of pain and other problems, whether physical, psychosocial or spiritual” (29). This generic definition of palliative care was used because a consensus definition of palliative psychiatry is not yet available. Additionally, each of the two case vignettes was accompanied by five items (see Table 1 for the case vignettes and Supplementary Material for the complete list of items). In each instance, participants responded on a 7-point Likert scale ranging from 1 (*not important/strongly disagree*) to 7 (*very important/strongly agree*), with a midpoint at 4 (*moderately important/neutral*).

**Table 1:** Case vignettes.

**Patient 1:**
−33-year-old male
- Schizophrenia with onset at age 17; no significant comorbidities
- Positive symptoms: auditory and visual hallucinations, persecutory delusions
- Negative symptoms: apathy, social withdrawal, poverty of speech (all rated severe)
Despite long-lasting high-dose pharmacological treatment (several atypical neuroleptics, haloperidol, clozapine, and their combinations), as well as electroconvulsive therapy, the patient has never been free from positive or negative symptoms. Multiple psychotherapies employing various approaches have also failed to stabilize the patient or to improve his quality of life. He does not wish to continue treatment because he feels it is too intrusive. While the positive symptoms predominated in the years immediately following his initial diagnosis, he developed severe negative symptoms, as well as aggression and self-injurious behavior, including burning himself with cigarettes. The negative symptoms and his strong functional deficits are exacerbated by chronic unemployment and an inability to live independently. Additionally, the patient has no family system, and his persisting illness has left him completely isolated, with no social contacts and no hobbies or interests. Two experts have declared that he possesses decision-making capacity regarding his illness and its treatment.
**Patient 2**:
−40-year-old male
- Recurrent major depressive disorder; no significant comorbidities
- Somatic symptoms: energy loss, insomnia, and fatigue
- Persistent suicidal ideation over the past 20 years; current acute and concrete suicidal intent
The patient underwent different forms of intensive, long-term, evidence-based psychotherapy, including specialized approaches such as cognitive behavioral analysis system of psychotherapy (CBASP) and interpersonal psychotherapy (IPT). Both psychotherapy alone and in combination with adequate treatment trials of antidepressants [selective serotonin reuptake inhibitors, tricyclic antidepressants, venlafaxine, augmentation with lithium and antipsychotic medications (quetiapine and aripiprazole)] failed to improve his depression, and the patient experienced significant adverse effects from several of the medications. Exhausted, he has decided to undergo electroconvulsive therapy as a last resort. However, maintenance electroconvulsive therapy again proved insufficient to prevent the reappearance of suicidal ideation; indeed, his symptoms worsened. Experiencing severe hopelessness, the patient states that his quality of life is very poor, that he doesn't want to deal with his illness anymore, and that he plans to commit suicide in the near future. Two experts have declared that he possesses decision-making capacity regarding his illness and its treatment.

*Case vignettes modified from Brenner et al. (28) and Baweja et al. (30) and adapted in the style of Trachsel et al. (9), Hodel et al. (26), and Stoll et al. (27)*.

### Ethics Approval

An ethics application was submitted to and approved by the Government Medical College, Thiruvananthapuram (HEC.No.01/06/2020/MCT, dated 07.02.2020).

### Statistical Analysis

Arithmetic means were calculated for the age and work experience items. Percentages were calculated for gender data, and for items related to treatment of patients with SPMI, palliative psychiatry in SPMI, and the two case vignettes. For better readability and to facilitate interpretation, 7-point Likert scale data were combined into three categories: *disagree/unimportant* (1–3), *neutral* (4), and *agree/important* (5–7).

To compare the samples from India and Switzerland, data from the respective samples were first tested for normal distribution. As the Shapiro-Wilks test indicated that all items deviated significantly from the normal distribution (*p* < 0.05), these differences were evaluated using the non-parametric Mann-Whitney *U* test, equivalent to Wilcoxon's rank-sum test. Mean and median values were calculated for each item, as the median is more useful for interpreting non-normally distributed data (31). In addition, the effect size *r* was determined to further refine interpretation of the data (31, 32). For increased readability, we report only significant comparisons with at least medium effect size (*r* ≥ 0.3) in the text. IBM SPSS Statistics Version 25 was used to perform the statistical analysis.

## Results

The fully completed questionnaires (*n* = 206) represented a response rate of 6.7%. Of these, 33% were women and 67% were men, with a mean age of 43.1 years (SD = 12.9, range = 25–78 years) and mean career duration of 19.0 years since graduation (SD = 12.9, range = 2–56 years).

### General Views on Treatment of Patients With SPMI

**Figure 1:**
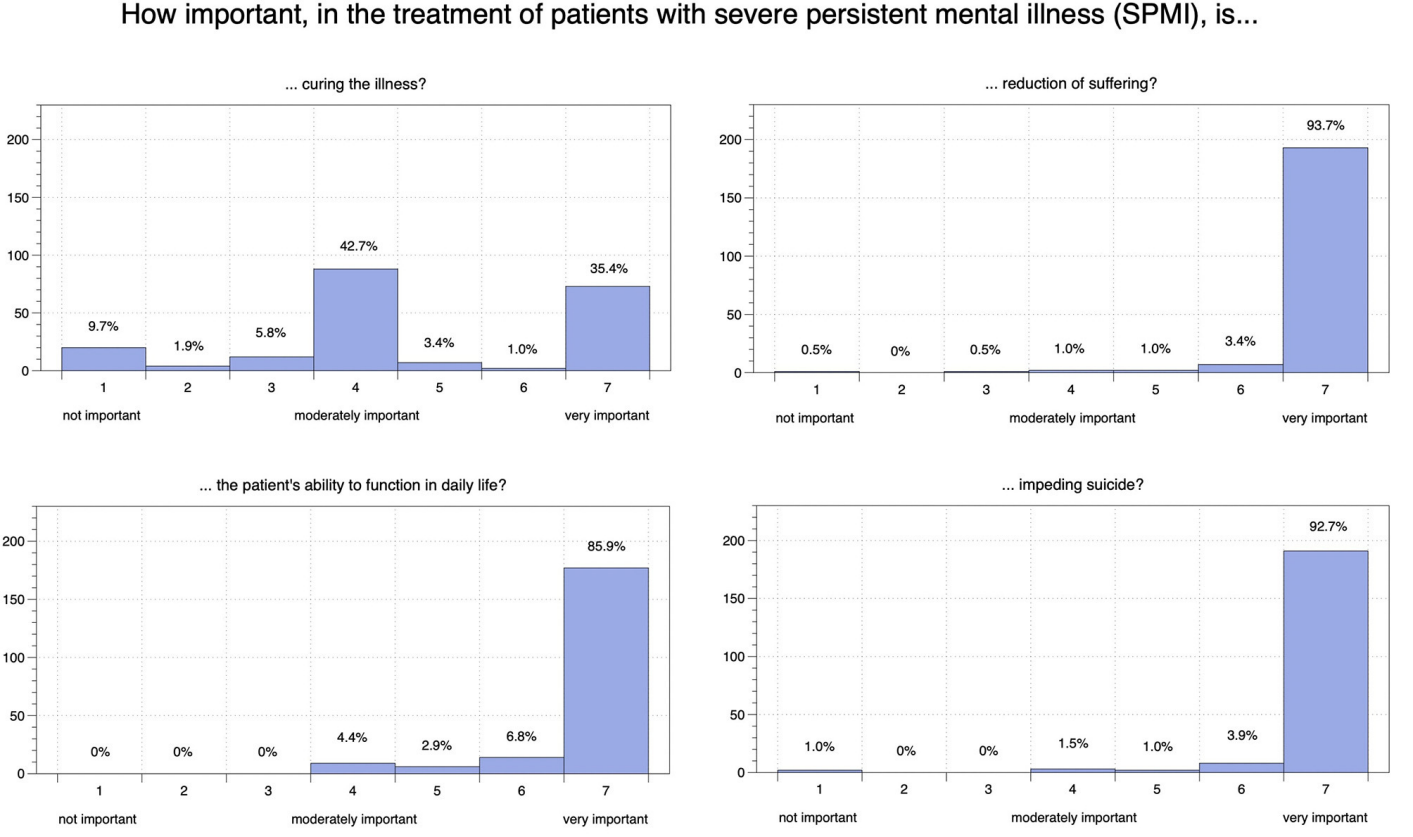
Indian psychiatrists' attitudes on general treatment of patients with SPMI.

Most participants (42.7%) felt it was moderately important to *cure* patients with SPMI; 39.8% regarded this as (very) important while 17.5% considered it less important to cure the illness (see Figure 1). Overwhelming majorities considered it (very) important to *reduce suffering* in patients with SPMI (98.1%), to help them *function in daily life* (95.6%), and to *impede suicide* (97.6%).

### General Views on Palliative Care and Its Applicability to Different Forms of SPMI

48.1% of respondents disagreed that the term *palliative* relates directly to end of life while 25.7% agreed and 26.2% were neutral. Regarding the proposition that *palliative care is indicated for some patients with SPMI*, 78.2% agreed while 16.5% remained neutral and only 5.3% disagreed. Regarding the proposition that *palliative care models are an important means of providing optimal care for patients with non-terminal illnesses*, 79.6% agreed while 13.1% remained neutral and 7.3% disagreed. Regarding the proposition that SPMI can be a terminal illness, 35.0% disagreed while 34.5% remained neutral and 30.6% agreed; a further 26.7% strongly disagreed, and 19.9% strongly agreed.

**Figure 2:**
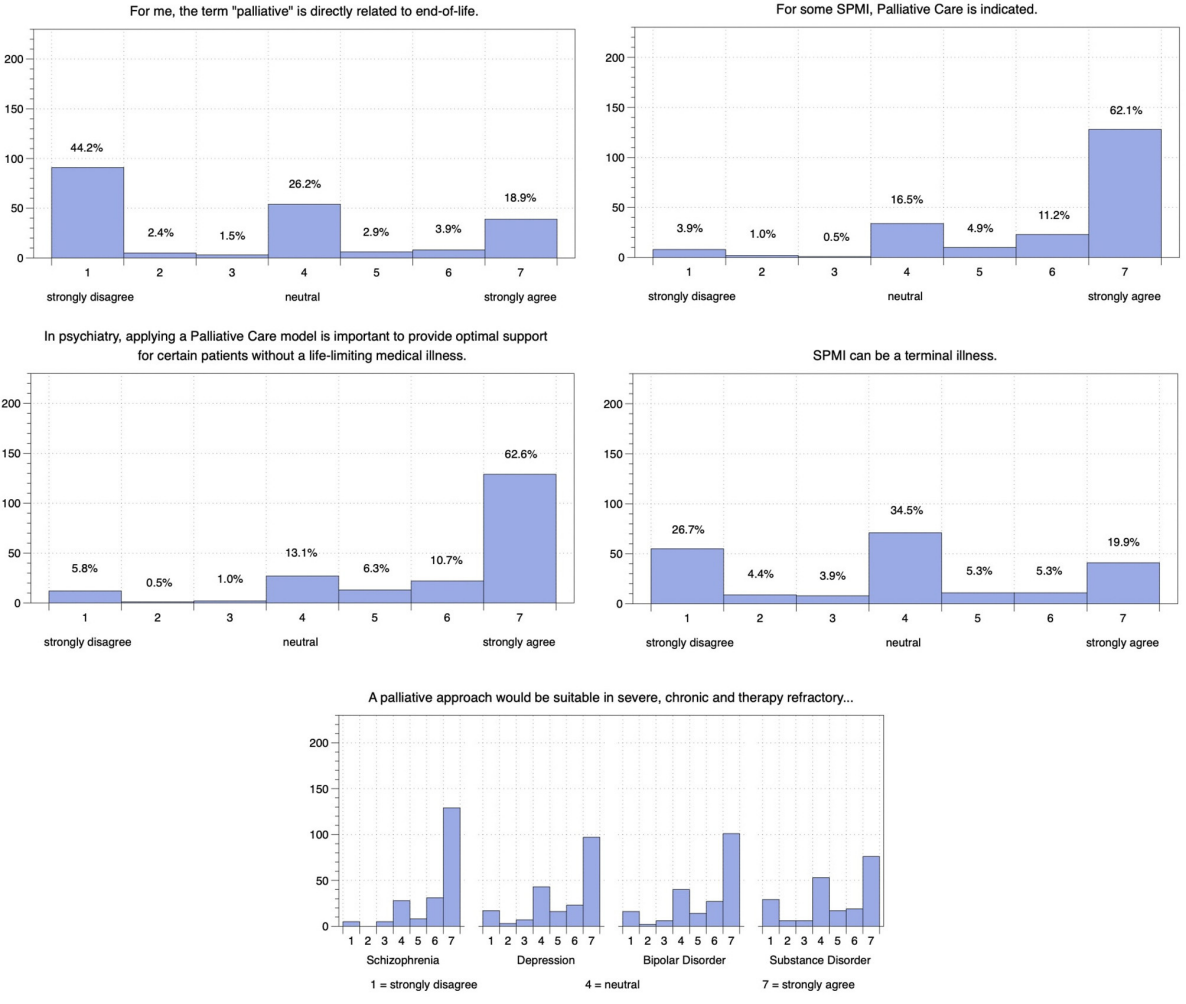
Indian psychiatrists' attitudes on palliative psychiatry and its use in patients with different SPMI.

Most participants (81.6%) agreed that in severe, chronic, and therapy-refractory schizophrenia a palliative approach would be suitable with just 4.9 % disagreeing (see Figure 2). The view that a palliative approach would be appropriate in cases of severe, chronic, and therapy-refractory bipolar disorder was shared by 68.9%, by 66.0% in cases of severe, chronic, and therapy-refractory depression, and by 54.4% in cases of severe, chronic, and therapy-refractory substance disorder with 11.7, 13.1, and 19.9 % disagreeing, respectively.

### Responses to the Case Vignettes

#### Schizophrenia

**Figure 3:**
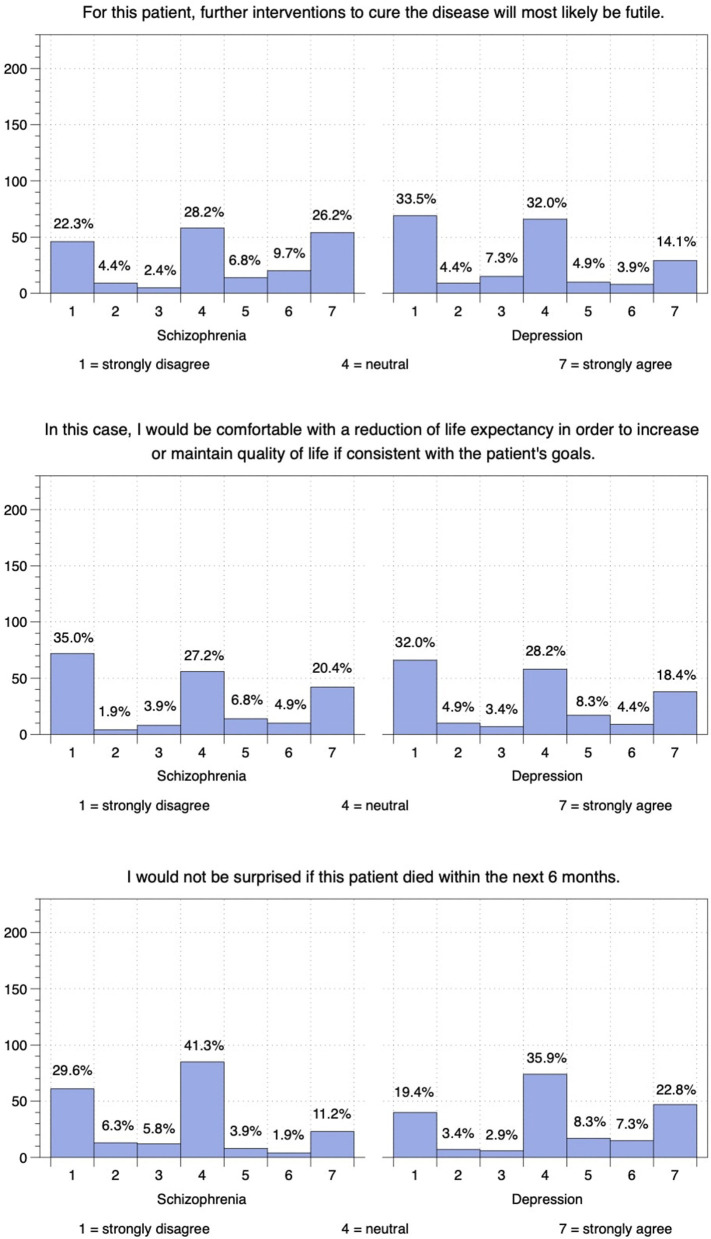
Indian psychiatrists' attitudes on the case vignettes.

Overall, 42.7% of respondents agreed that *further curative intervention would probably be futile in this case of schizophrenia* while 29.1% disagreed and 28.2% remained neutral (see Figure 3). 40.8% indicated that they would not be comfortable with *a reduction in life expectancy to increase or maintain the patient's quality of life* while 32.0% indicated they would be comfortable with this and 27.2% remained neutral. When asked whether they would be *surprised if the patient died within the next 6 months*, 41.7% agreed while 41.3% remained neutral and only 17.0% disagreed.

#### Recurrent Major Depressive Disorder

Most respondents (45.1%) disagreed that *further intervention to cure the patient's depression would probably be futile* while 32.0% remained neutral and 22.8% agreed (see Figure 3). While 40.3% would not be comfortable with *a reduction of life expectancy to increase or maintain the quality of life of this patient*, 31.1% said they would be, and 28.2% remained neutral. Finally, 38.3% of respondents said they would not be *surprised if the patient died within the next 6 months* while 35.9% remained neutral and 25.7% said they would be surprised.

### Comparison of Psychiatrists' Views in India and Switzerland

Psychiatrists in India agreed significantly less than psychiatrists in Switzerland that *SPMI can be a terminal illness* (*U* = 16244.0, *p* < 0.001, *r* = −0.55; see Table 2), and considered it significantly more important to *impede suicide* when treating SPMI (*U* = 21310.0, *p* < 0.001, *r* = 0.48; see Table 2). However, psychiatrists in India agreed significantly more than psychiatrists in Switzerland that *a palliative approach would be appropriate for patients with severe, chronic, and therapy-refractory schizophrenia* (*U* = 28897.5, *p* < 0.001, *r* = 0.32).

**Table 2:** Comparison of India and Switzerland.

**Item**	**Group**	***n***	***M***	***Mdn***	***U***	***Z***	***p-value***	***r***
Ia: curing the illness	India	206	4.73	4				
	Switzerland	447	3.73	4				
	Total	653			32431.0	−6.27	<0.001***	−0.25
Ib: reduction of suffering	India	206	6.87	7				
	Switzerland	456	6.66	7				
	Total	662			38091.5	−5.64	<0.001***	−0.22
Ic: function in daily life	India	206	6.74	7				
	Switzerland	456	6.55	7				
	Total	662			39209.0	−4.42	<0.001***	−0.17
Ie: impeding suicide	India	206	6.84	7				
	Switzerland	454	5.80	6				
	Total	660			21310.0	−12.38	<0.001***	−0.48
If: term “palliative”	India	206	3.29	4.00				
	Switzerland	452	4.24	4.00				
	Total	658			34116.0	−5.58	<0.001***	−0.22
Ig: SPMI and palliative care	India	206	6.00	7.00				
	Switzerland	444	5.39	6.00				
	Total	650			31241.5	−6.75	<0.001***	0.26
Ih: Palliative care support (not life-limiting)	India	206	5.96	7.00				
	Switzerland	449	5.43	6.00				
	Total	655			32133.5	−6.53	<0.001***	0.26
Ii: SPMI can be terminal	India	206	3.83	4.00				
	Switzerland	453	6.36	7.00				
	Total	659			16244.0	−14.22	<0.001***	−0.55
Ij: schizophrenia (palliative approach)	India	206	6.12	7.00				
	Switzerland	452	5.24	6.00				
	Total	658			28897.5	−8.10	<0.001***	0.32
Ik: depression (palliative approach)	India	206	5.40	6.00				
	Switzerland	452	5.00	6.00				
	Total	658			37811.0	−3.96	<0.001***	0.15
Il: bipolar disorder (palliative approach)	India	206	5.52	6.00				
	Switzerland	452	4.94	6.00				
	Total	658			35209.0	−5.14	<0.001***	0.20
Im: substance disorder (palliative approach)	India	206	4.86	5.00				
	Switzerland	452	5.26	6.00				
	Total	658			43921.5	−1.19	0.233	−0.05
(1) Schizophrenia								
IIb**:** futility of further intervention	India	206	4.27	4				
	Switzerland	448	4.82	5				
	Total	654			40541.5	−2.53	0.011*	−0.10
IIc: quality of life vs. reduction of life expectancy	India	206	3.65	4				
	Switzerland	448	5.15	5				
	Total	654			28752.0	−7.88	<0.001***	−0.31
IIe: dying within the next 6 months (surprise question)	India	206	3.34	4				
	Switzerland	450	4.39	4				
	Total	656			31333.5	−6.80	<0.001***	−0.27
(2) Depression								
IIb: futility of further intervention	India	206	3.38	4				
	Switzerland	450	4.41	5				
	Total	656			32672.0	−6.14	<0.001***	−0.24
IIc: quality of life vs. reduction of life expectancy	India	205	3.63	4				
	Switzerland	450	5.05	5				
	Total	655			28731.5	−7.87	<0.001***	−0.31
IIe: dying within the next 6 months (surprise question)	India	206	4.23	4				
	Switzerland	450	5.71	6				
	Total	656			27121.5	−8.76	<0.001***	−0.34

Only abbreviated questionnaire items are shown [see Supplementary Material for complete list of items]. r: effect size [for calculation see citations (31, 32)]. Significant p values (p <0.05) in bold: ***p <0.001, *p <0.05.

Regarding the case vignette of a patient with severe and persistent schizophrenia, psychiatrists in India were significantly less comfortable than psychiatrists in Switzerland with a *reduction in life expectancy to increase or maintain quality of life* (*U* = 28752.0, *p* < 0.001, *r* = −0.31; see Table 2). The same was true in the case of the patient with recurrent major depressive disorder (*U* = 28731.5, *p* < 0.001, *r* = −0.31). In this case, psychiatrists in India also reported to a significantly greater extent that they would be surprised *if the patient died within the next 6 months* (*U* = 27121.5, *p* < 0.001, *r* = 0.34).

## Discussion

For a vast majority of psychiatrists in India, suicide prevention in patients with severe and persistent mental illness (SPMI) was very important. Psychiatrists in India also tended not to view SPMI as a terminal illness with 26.7% even strongly disagreeing with this notion. However, curing the illness was not very important for the majority, and some psychiatrists in India even regarded further curative treatment as futile in specific cases. Almost all psychiatrists emphasized the importance of reducing suffering and improving functionality of SPMI patients in everyday life, both of which are central concepts in palliative psychiatry (8, 10) as a genuine biopsychosocial approach which systematically integrates biological, psychological, social, and existential factors of care (12, 13). Consecutively, a majority believed that palliative psychiatry is indicated for some patients with SPMI (especially schizophrenia), even in the absence of a life-limiting somatic disease. However, when confronted with vignettes of specific patients with severe, chronic, and therapy-refractory schizophrenia and depression, most psychiatrists in India indicated that they would not be comfortable with improving quality of life at the expense of life expectancy.

At first glance, this strong emphasis on both duration and quality of life of SPMI patients may be difficult to reconcile. However, palliative psychiatry can be accommodated alongside a curative approach, and as the disorder does not need to be terminal for the application of palliative psychiatry (8), it does not necessarily mean discontinuing curative treatment (6). In line with this interpretation, only a minority of surveyed psychiatrists in India found that the term palliative directly relates to end of life.

### Comparison of Psychiatrists' Attitudes in India and Switzerland

The participating psychiatrists in India tended to support both curative and palliative approaches for patients with SPMI more strongly than psychiatrists in Switzerland. Regarding curative approaches, psychiatrists in India considered it more important to impede suicide and to cure patients with SPMI than psychiatrists in Switzerland. In line with these attitudes, psychiatrists in India were less likely to believe that SPMI can become a terminal illness. The same trend is apparent in both case vignettes; psychiatrists in India would be more surprised if the patient with severe and persistent schizophrenia or recurrent major depressive disorder would die within the next 6 months. They were less likely to consider further intervention futile in both cases than psychiatrists in Switzerland, and they would not be comfortable with a reduction of life expectancy in either case, even at the expense of quality of life.

How might we explain the stronger support for curative approaches and suicide prevention in SPMI of psychiatrists in India? First, as referred to in the introduction, it is considered important not to classify patients as chronic or therapy-refractory because of insufficient treatment and resources; on that basis, a curative approach should not be abandoned (6). As psychiatrists in India are likely very aware of this issue, they may therefore tend to favor a curative approach even for patients classified as suffering from chronic, severe, and therapy-refractory mental disorders.

Second, although suicide rates in India are generally comparable to Switzerland (33), in persons aged between 15 and 49, suicide rates in India are almost twice as high as in Switzerland (34). Vijayakumar (35) reported that more than 70% of suicides in India involve persons younger than 44, which is the age range in the case vignettes. In a comparative study of attitudes to suicide among medical students in India and Austria, overall attitudes were more negative in India, and suicide was associated with mental illness, cowardice, and even illegality (36). In India, attempted suicide was only recently decriminalized in the Mental Health Care Act of 2017 (37). Indian medical students also exhibit a strong aversion to physician-assisted suicide (36). In contrast, physician-assisted suicide has been legal for decades in Switzerland, and the psychiatrists surveyed in Switzerland supported the idea for patients with SPMI to some extent (26).

Third, while it might seem interesting to explore whether these differences in pro-life attitude relate to religious beliefs, Etzersdorfer et al. (36) found no evidence that religion played a role in the differing attitudes to suicidal behavior of medical students from India and Austria. Referring primarily to the Hindu religion, they found no greater aversion to suicide than in the Christian religion and further noted that there is some evidence of institutionalized suicide in India. In a more recent questionnaire study, Thimmaiah et al. (38) reported that negative attitudes to suicidality are less common among Hindus than Muslims, and these cultural differences invite further research.

Besides the greater support for curation and suicide prevention, psychiatrists in India also assigned greater importance to the reduction of suffering and functionality in daily life than their counterparts in Switzerland. They agreed more strongly that palliative approaches might be indicated in patients with SPMI, even in the absence of life-limiting disease.

By implication, the participating psychiatrists in India tended to support both curative and palliative approaches for patients with SPMI. This suggests that, for psychiatrists in India, curative approaches and palliative psychiatry are not mutually exclusive but can complement each other to alleviate suffering and increase functionality in daily life in parallel to curative treatments (8). Such a notion of compatibility of palliative psychiatry and curative approaches may be facilitated by regarding the term *palliative* as not directly related to the end of life, which psychiatrists in India were significantly more likely to do than psychiatrists in Switzerland.

### Strengths and Limitations of the Study

One limitation of the study is the low response rate of 6.7% in the Indian sample (compared to a response rate of 34.9% in the Swiss sample). Basing the calculation on the population who clicked on the link yields a response rate of 36.7%. The generalizability of the data may therefore be limited as the participants are likely to have an existing interest in SPMI and palliative care. However, there is evidence that non-response bias may be of less concern in physician surveys than in surveys of other populations (39). Also, response rates are known to be lower in online surveys (40) and in surveys of physicians (39), especially psychiatrists (41).

As only psychiatrists were surveyed, the generalizability of the response patterns to other professions is limited.

The observed differences between the two samples might relate to differences in age and career duration. It is also important to note that response behavior can vary across countries and cultures (42), which may be compounded by the fact that the questionnaires were presented in different languages (German and English). For example, the psychiatrists in India (up to 30%) chose the middle category more often than those in Switzerland. To limit and identify any interpretive bias associated with dichotomous significance testing, effect sizes were also calculated.

Other general limitations of this type of survey have already been mentioned in previous studies based on the same questionnaire (9, 26, 27) but can be briefly summarized as follows. First, a Likert scale can only reflect the opinions of individuals to a limited extent and cannot fully capture the complexity of the topic. Importantly, we did not assess how the individual participants conceptualize palliative psychiatry. Second, the case vignettes represent highly specific cases and are not representative of the respective disorders in general.

### Implications for Clinical Practice and Future Research

The hesitation to integrate palliative psychiatry in existing mental healthcare structures may reflect the fact that it is too often associated with end of life, giving up, and hopelessness (2, 3, 7). The present findings and particularly the views of psychiatrists in India suggest that first, palliative psychiatry is considered valuable across cultures as a means of improving patients' quality of life, without necessarily accepting a reduction in life expectancy, and second, rather than asking “palliative or curative?,” we should discuss the possibility of palliative and curative, combining both approaches to offer optimal treatment to patients with SPMI. As Strand and colleagues (7) have argued, “[…] the type of interventions referred to as palliative are by no means ‘novel' and ‘cutting-edge'—quite the contrary, we interpret palliative care as an approach defined by its goals and not by the use of specific treatments” (p. 6). It seems important, then, that researchers and clinicians focus on developing a framework for clinical practice that optimally combines curative and palliative approaches for the individual patient and situation.

## Data Availability Statement

The raw data supporting the conclusions of this article will be made available by the authors, without undue reservation.

## Ethics Statement

The studies involving human participants were reviewed and approved by Ethics Committee, Government Medical College, Thiruvananthapuram (HEC.No.01/06/2020/MCT, dated 07.02.2020). The patients/participants provided their written informed consent to participate in this study.

## Author Contributions

AM, CV, AP, and MT conceived the study and were involved in adapting the questionnaire for data collection in India. AM, CV, JS, and MT were involved in data collection. JS, MT, and AW evaluated the data and drafted the article. All authors were involved in critical revision of the draft manuscript and all approved the final version submitted for publication.

## Funding

The Swiss arm of the study was supported by the Palliative Care Research funding program of the Swiss Academy of Medical Sciences (SAMS), the Gottfried and Julia Bangerter-Rhyner Foundation, and the Stanley Thomas Johnson Foundation. None of the funding bodies influenced the authors in writing this manuscript.

## Author Disclaimer

The views expressed here do not necessarily represent the views of the funding agencies.

## Conflict of Interest

The authors declare that the research was conducted in the absence of any commercial or financial relationships that could be construed as a potential conflict of interest.

## Publisher's Note

All claims expressed in this article are solely those of the authors and do not necessarily represent those of their affiliated organizations, or those of the publisher, the editors and the reviewers. Any product that may be evaluated in this article, or claim that may be made by its manufacturer, is not guaranteed or endorsed by the publisher.
