# Supplementary material for: Palliative Psychiatry for Patients With Severe and Persistent Mental Illness: A Survey on the Attitudes of Psychiatrists in India Compared to Psychiatrists in Switzerland
Source: Front Psychiatry. 2022 May 26;13:858699. doi: 10.3389/fpsyt.2022.858699 (PMC9178077; doi:10.3389/fpsyt.2022.858699)
Supplement: Supplementary file 1 [file Table_1.DOCX]

Supplementary Material

**Table S1**

*Survey items*

| **I: Questions regarding the treatment of patients with severe and persistent mental illness (SPMI)** | |
| --- | --- |
| In the treatment of patients with SPMI, how important are the following: | |
|  | a) curing the illness |
|  | b) reduction of suffering |
|  | c) patient’s ability to function in daily life |
|  | d) patient retaining decision making autonomy |
|  | e) impeding suicide |
| **According to the World Health Organization, palliative care “is an approach that improves the quality of life of patients and their families facing problems associated with life-threatening illness through the prevention and relief of suffering by means of early identification and impeccable assessment and treatment of pain and other problems, physical, psychosocial and spiritual.”** | |
| How strongly do you agree or disagree with the following statements? | |
|  | f) For me, the term “palliative” relates directly to end-of-life. |
|  | g) For some SPMI, palliative care is indicated. |
|  | h) In psychiatry, it is important to apply a palliative care model to provide optimal support for certain patients with no life-limiting medical illness. |
|  | i) SPMI can be terminal. |
|  | j) In severe, chronic, and therapy-refractory *schizophrenia*, a palliative approach would be appropriate. |
|  | k) In severe, chronic, and therapy-refractory *depression*, a palliative approach would be appropriate. |
|  | l) In severe, chronic, and therapy-refractory *bipolar disorder*, a palliative approach would be appropriate. |
|  | m) In severe, chronic and therapy-refractory *substance disorder*, a palliative approach would be appropriate. |
| **II: Case vignettes:** Please provide an evaluation of the case vignettes. | |
|  | a) In this case, I would not proceed against the patient’s wishes. |
|  | b) For this patient, any further intervention to cure their schizophrenia/depression would most likely prove futile. |
|  | c) In this case, I would be comfortable with a reduction of life expectancy in order to increase or maintain the patient’s quality of life (if consistent with their goals). |
|  | d) In this case, I would accept a temporary decrease in quality of life as a consequence of coercive measures. |
|  | e) I would not be surprised if this patient died within the next 6 months. |

*Note.* The Table has been designed in the style of and adapted after Trachsel et al., Hodel et al., and Stoll et al. (9, 25, 26).
